# Characteristic analysis of epileptic brain network based on attention mechanism

**DOI:** 10.1038/s41598-023-38012-0

**Published:** 2023-07-03

**Authors:** Hong-Shi Yu, Xiang-Fu Meng

**Affiliations:** 1 School of Electronics and Information Engineering, Liaoning Technical University, Huludao, 125105 China; 2 Liaoning Key Laboratory of Radio Frequency Big Data Intelligent Application, Huludao, 125105 China

**Keywords:** Physiology, Diseases

## Abstract

Constructing an efficient and accurate epilepsy detection system is an urgent research task. In this paper, we developed an EEG-based multi-frequency multilayer brain network (MMBN) and an attentional mechanism based convolutional neural network (AM-CNN) model to study epilepsy detection. Specifically, based on the multi-frequency characteristics of the brain, we first use wavelet packet decomposition and reconstruction methods to divide the original EEG signals into eight frequency bands, and then construct MMBN through correlation analysis between brain regions, where each layer corresponds to a specific frequency band. The time, frequency and channel related information of EEG signals are mapped into the multilayer network topology. On this basis, a multi-branch AM-CNN model is designed, which completely matches the multilayer structure of the proposed brain network. The experimental results on public CHB-MIT datasets show that eight frequency bands divided in this work are all helpful for epilepsy detection, and the fusion of multi-frequency information can effectively decode the epileptic brain state, achieving accurate detection of epilepsy with an average accuracy of 99.75%, sensitivity of 99.43%, and specificity of 99.83%. All of these provide reliable technical solutions for EEG-based neurological disease detection, especially for epilepsy detection.

## Introduction

Epilepsy is a worldwide nervous system disease caused by sudden abnormal discharge of neurons. According to statistics, 50 million people worldwide are suffering from epilepsy. In recent years, the research on timely, accurate, and automatic epilepsy detection methods has attracted the attention of a large number of scholars. Taking the opportunity of studying epilepsy detection methods, this study aims to explore effective methods to depict brain states, design CNN architecture to enhance feature extraction, and provide valuable reference schemes for timely and accurate EEG-based nervous system disease detection.

So far, many epilepsy detection technologies have been developed. The most commonly used method of epilepsy detection is to analyze scalp electroencephalogram (EEG) signals, which are records of brain electrical activity. For example, Bhattacharyya and pachori^1^ combined multivariate empirical wavelet transform and well-known classifier to classify epileptic signals based on EEG. Zhang et al.^2^ used the FPE-complexity of EEG signals as input, and utilized extreme machine learning and support vector machine to detect epilepsy. Radman et al.^3^ combined the Dempster-Shafer Evidence Theory (DSET) and Ensemble Decision Tree (EDT) to conduct EEG-based epilepsy detection. In addition, brain is an extremely complex time series system with obvious nonlinearity and instability. The information from EEG signals constantly changes in both the time and frequency domains, and has strong correlation between brain electrodes (channels). The frequency and spatiotemporal information of EEG signals codetermine the brain state. Therefore, exploring effective fusion methods of multiple information (frequency, time, and brain regions) is the key to analyzing brain features in epileptic states and improving the accuracy of epilepsy detection^4–6^.

In recent years, the theory of complex networks has made significant progress^7–9^. Complex network is composed of nodes and edges connecting two nodes. It has been proven that complex networks are effective tools for analyzing complex systems from the perspective of network topology, which abstractly describes the specific behaviors of the studied system. Time series complex networks are an important branch of complex network theory. By constructing a network of time series, the complex features contained therein can be mapped to the network topology, thereby enabling the characterization of complex systems. Constructing time series complex network (brain network) related to brain behavior is one of the important ways to analyze the state of the brain ^10–12^. Specifically, the brain network can be constructed from multi-channel EEG signals, and then the brain state can be explored through network topology analysis. In general, the brain network can be inferred by setting the brain electrodes (channels) as nodes, and then the edges between the nodes can be determined by various related metrics. Moreover, multilayer network are the latest development of complex network theory^13–15^. The multilayer network has multiple layers and can describe different aspects of the studied system. The multilayer structure makes it possible to describe complex systems more comprehensively and accurately. Some successful applications of multilayer networks can be found in the fields of chemical systems^16^, EEG signal analysis^17, 18^ and traffic network analysis^19^.

The human brain has obvious multi-frequency characteristics. When multilayer network is introduced into brain research, spatiotemporal characteristics in each frequency band can be mapped into a single layer. MMBN considers the specific information of multiple frequency bands and can be used as an effective feature for epilepsy detection. However, it should be noted that in the analysis process, multilayer network are usually represented as a series of adjacency matrices. Each adjacency matrix corresponds to a single layer. In the face of such multidimensional samples, traditional classifiers, such as support vector machines, cannot be directly used for classification. As the most advanced theory in machine learning, deep learning^20–23^ has received extensive and continuous attention. Specifically, deep learning is an end-to-end learning framework that can extract deeper internal representations from the input itself^24–27^. So far, deep learning has shown great potential in epilepsy research. For instance, Li et al.^28^ proposed a deep learning method combining the fully convolution network and the long short-term memory (LSTM) to automatically detect epilepsy. Specifically, the full connection layer network and LSTM are used to extract EEG-based epileptic features and explore the inherent temporal correlation in EEG signals. Kemal et al.^29^ developed a stacking ensemble method to detect epilepsy using five deep neural network (DNN) models. Gao et al.^30^ used approximate entropy and recursive quantitative analysis to extract the features of EEG signals, and then establishes convolutional neural networks to detect epilepsy. Zhao et al.^31^ developed a novel one-dimensional CNN model to detect epilepsy with raw EEG signals. The model used three convolution blocks including BN layer and dropout layer for feature extraction. Naseem et al.^32^ employed a method integrating CWT and CNN to classify EEG data and detect seizures caused by epilepsy and brain tumors. The results show that the deep learning model is conducive to EEG classification and timely prediction of seizures to avoid damage caused by repeated seizures. Some researchers tried to add the attention mechanism module to the CNN model^33–35^. The attention mechanism introduces weight on the basis of the original model, and can help the new model focus on informative and important features.

**Figure 1:**
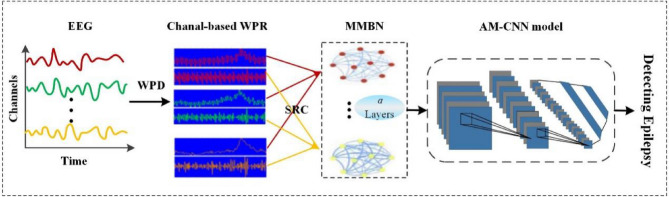
Overall framework of research work. WTP means wavelet packet transform, WPR means wavelet packet reconstruction and SRC means Spearman rank correlation coefficient.

Motivated by the above-described background and progress, we propose an epilepsy detection method combining multilayer brain network and deep learning. In detail, the EEG signals of each channel are decomposed into multiple frequency bands through wavelet packet decomposition and reconstruction. Then, constructing MMBN, where each layer is a spatiotemporal feature topology of EEG signals in a specific frequency band. Compared with single-layer network, this multilayer brain network integrates the information from multiple frequency bands, helping to provide a more comprehensive description of brain states. In addition, considering the strong ability of deep learning to learn structural features, we carefully developed a CNN model based on attention mechanism (AM-CNN) with MMBN as input. Through evaluation on the CHB-MIT dataset, this method achieved excellent performance with an accuracy of 99.75%. The results indicate that this study can effectively characterize the brain state during seizures, extract essential features for precise classification, and is expected to provide reference for other EEG signal based neurological disease detection. The overall structure of our work is shown in Fig. 1.

## Results

### MMBN analysis of brain-topological characteristic of epileptic

We randomly select eight subjects and calculate the measurement statistics of the MMBN obtained from the normal state and the seizure state respectively, and then *t*-test was performed on them. Four network measures are introduced, including average clustering coefficient $\overline{C}$, clustering coefficient entropy $E_{C}$, spectral radius $R$ and graph energy $E$. These statistical measures are defined as the following equations:

1 $$\overline{C} = \frac{1}{N}\sum\limits_{\nu = 1}^{N} {C\left( \nu \right)}$$

where $N$ is the total number of nodes (channels) in the network, $C\left( \nu \right)$ means clustering coefficient of node $\nu$, the mathematical expression is

2 $$C\left( \nu \right) = \frac{{2t_{\nu } }}{{k_{\nu } (k_{v} - 1)}} = \frac{{\sum\nolimits_{\kappa ,\alpha } {w_{\nu \kappa } w_{\nu \alpha } w_{\kappa \alpha } } }}{{k_{\nu } (k_{v} - 1)}}$$

3 $$k_{\nu } = \sum\nolimits_{\kappa \ne \nu } {w_{\nu \kappa } }$$

where $t_{\nu }$ is the total number of closed triangles containing node $\nu$, $k_{\nu }$ is the degree of node $\nu$, $w_{\nu \kappa }$ represents the edge weight between nodes $\kappa$ and $\nu$, $w_{\nu \alpha }$ represents the edge weight between nodes $\alpha$ and $\nu$, $w_{\kappa \alpha }$ represents the edge weight between nodes $\kappa$ and $\alpha$.

4 $$E_{C} = - \sum\limits_{\nu = 1}^{N} {P_{C} } \left( \nu \right)\log P_{C} \left( \nu \right)$$

where $P_{C} \left( \nu \right)$ is expressed as

5 $$P_{C} \left( \nu \right) = {{C\left( \nu \right)} \mathord{\left/ {\vphantom {{C\left( \nu \right)} {\sum\limits_{\nu = 1}^{N} {C\left( \nu \right)} }}} \right. \kern-0pt} {\sum\limits_{\nu = 1}^{N} {C\left( \nu \right)} }}$$

6 $$R = \mathop {\max }\limits_{\nu } \left( {\left| {\lambda_{v} } \right|} \right)\begin{array}{*{20}c} {} & {\nu = 1,2, \ldots ,N} \\ \end{array}$$

where $\lambda_{\nu }$ denotes the $\nu {\text{-th}}$ eigenvalue of the adjacency matrix(single-layer network).

7 $$E = \sum\limits_{\nu = 1}^{N} {\left| {\lambda_{\nu } } \right|} \begin{array}{*{20}c} {} & {\nu = 1,2, \ldots ,N} \\ \end{array}$$

**Figure 2:**
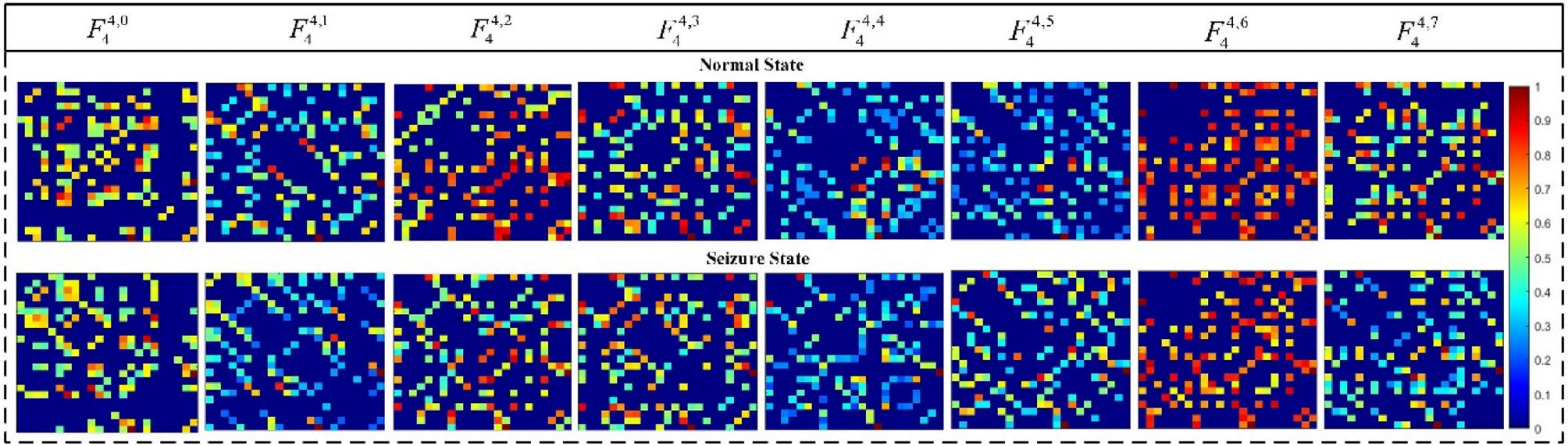
MMBN for normal state (upper row) and seizure state (lower row).

It can be seen from Table 1 that 93.75% of *p*-value values are less than 0.001, and 100% of *p*-value values are less than 0.05. Figure 2 shows two randomly selected 8-layer brain networks. One sub-graph corresponds to one layer of the MMBN and is related to a specific frequency band. The MMBN in the normal state is located in the upper row, and the MMBN in the seizure state is located in the lower row. As can be seen, the network topology shows obvious differences in different frequency bands. All the above indicates that the proposed multilayer brain network can effectively characterize the differences in the topology of brain networks between seizure and normal, and confirms the importance of frequency and electrodes (channels) in epilepsy detection research.

**Table 1:** *p*-value of MMBN statistical measure between normal state and seizure state $(* :p < 0.05, * * :p < 0.001)$.

	Sub_1	Sub_2	Sub_3	Sub_4	Sub_5	Sub_6	Sub_7	Sub_8
$\overline{C}$	**	**	*	**	**	**	**	**
$E_{{\text{C}}}$	**	**	**	**	*	**	**	**
$E$	**	**	**	**	**	**	**	**
$R$	**	**	**	**	**	**	**	**

### Attention mechanism-based epilepsy detection

Taking MMBN that integrates spatiotemporal features across eight frequency bands as input, the AM-CNN model was trained using Keras through a fully supervised process and used to perform epilepsy detection on 18 selected subjects. The final results indicated that the epilepsy detection scheme combining multilayer brain network and AM-CNN model can effectively distinguish between normal state and epileptic state, with a classification accuracy of 99.75% sensitivity of 99.43%, and specificity of 99.83%. Some existing research results on the CHB-MIT dataset are also listed in Table 2. The proposed method is better than them in terms of accuracy and sensitivity, and is extremely close to Dang's work in terms of specificity but superior to other works. All of these provide new ideas for the characterization of EEG signals, and also provide technical support for the construction of an efficient and accurate epileptic state detection system.

**Table 2:** Epilepsy detection result of the proposed method versus some existing works on CHB-MIT.

Work	Year	Subject	Accuracy (%)	Sensitivity (%)	Specificity (%)
Zabihi et al.^36^	2016	23	94.69	89.10	94.80
Fergus et al.^37^	2016	23	93.00	88.00	88.00
Bhattacharyya et al.^1^	2017	23	99.41	97.91	99.57
Kaleem et al.^38^	2018	23	92.91	94.27	91.55
Ke et al.^39^	2018	18	98.13	98.85	97.47
Chen et al.^40^	2020	22	94.00	97.90	–
Li et al.^28^	2020	–	95.29	95.42	95.29
Dang et al.^41^	2021	18	99.56	99.29	99.84
This work	2022	18	99.75	99.43	99.83

## Discussions

### Effectiveness of MMBN

**Figure3:**
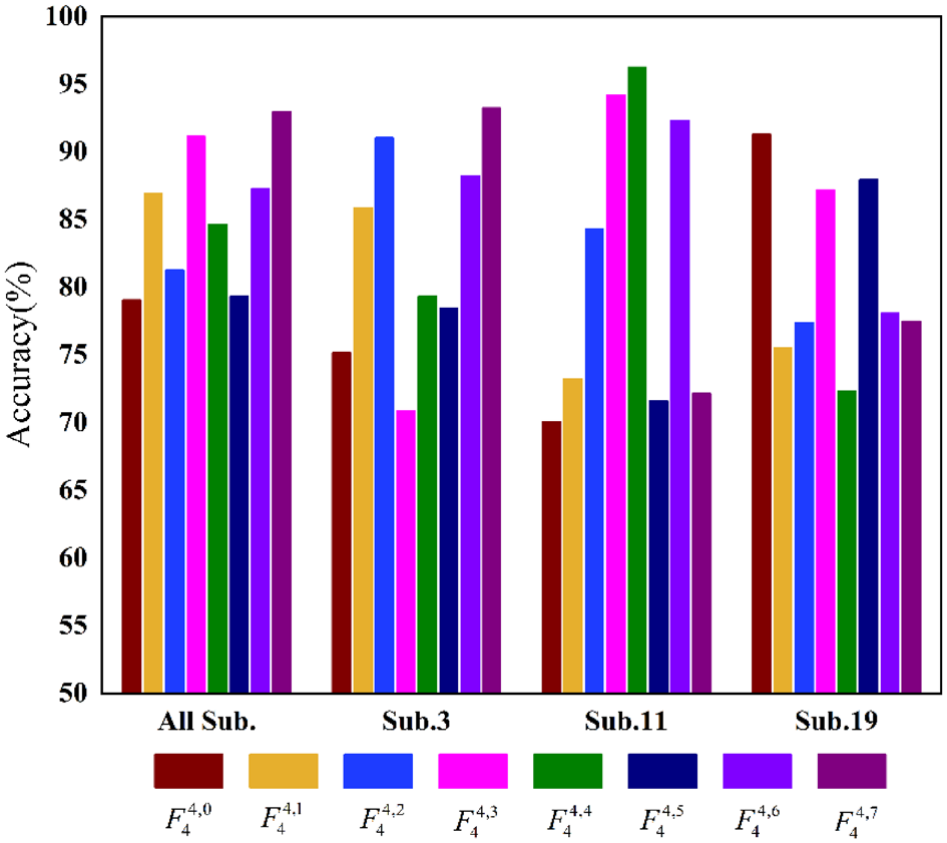
The contribution of different frequency bands in epilepsy detection (Sub.3, Sub.11, and Sub.19 are three randomly selected subjects).

The proposed method in this paper achieves an excellent epilepsy detection performance with an average accuracy of 99.75%. This reflects the overall effectiveness of using multilayer brain networks as new feature inputs. At the same time, we also analyze the effectiveness and contribution of each frequency band to epilepsy detection. Specifically, taking a single-layer network as input, epilepsy detection is performed on 18 subjects using the same AM-CNN architecture. The results are shown in Fig. 3. The average detection accuracy ranges from 79.06% to 92.97%. This indicates that all eight frequency bands divided in this study are helpful for epilepsy detection, but the contribution of each frequency band is different. Meanwhile, the contributions of each frequency band are also influenced by individual differences among the subjects. Taking frequency band $F_{4}^{4,3}$ as an example, the detection results of different subjects fluctuate between 70.9% and 94.2%. This further confirms that brain function has multi-frequency characteristics, and MMBN fused with information from multiple frequency bands can more effectively decode epileptic brain states.

### Effect of attention mechanism

In the methods section, AM-CNN architecture is designed to conduct feature extraction from multilayer brain networks. To further emphasize and illustrate the role of attention mechanism, we construct a comparison model to validate the effectiveness of the attention mechanism using the same inputs. The architecture and test results of comparative model is shown in Table 3. The performance of comparative model with accuracy of 93.54%, sensitivity of 93.18%, and specificity of 92.76% is significantly lower than those of AM-CNN. The complete AM-CNN does achieve the better performance. This indicates that attention mechanisms play a crucial role in enhancing feature extraction processes in this study.

**Table 3:** Performance of the proposed AM-CNN model and comparative model.

	Model architecture	Accuracy (%)	Sensitivity (%)	Specificity (%)
Comparative Model	Remove attention paths (i.e. *L*3, *L*4, *L*5, and *L*6)	93.54	93.18	92.76
AM-CNN	See Fig. 5	99.75	99.43	99.83

### Rationality of frequency band division

**Figure 4:**
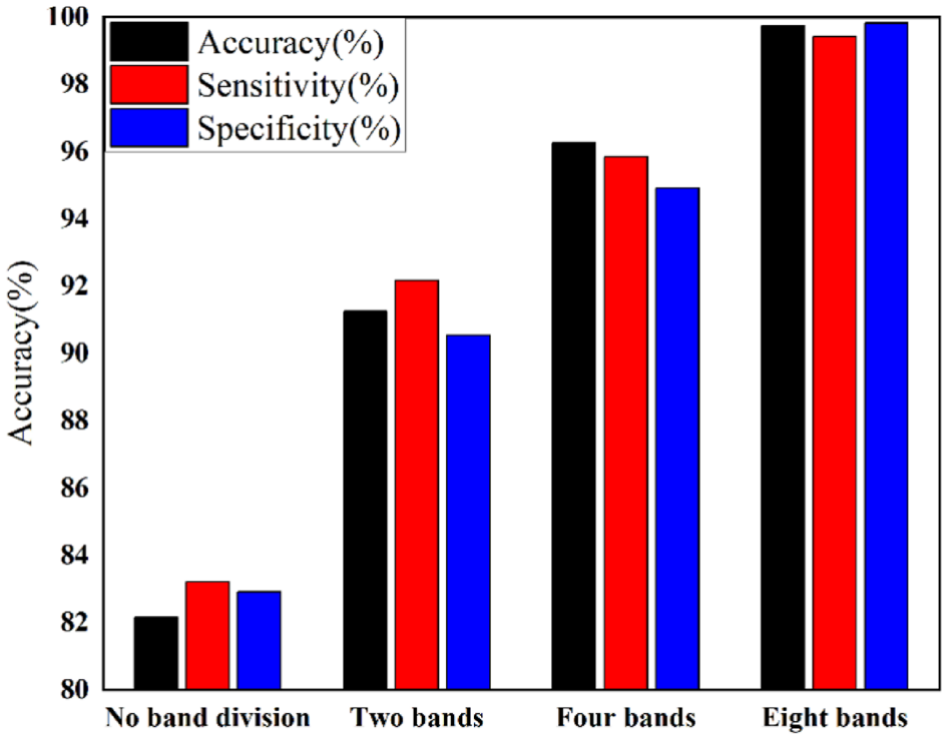
Epilepsy detection results by using different number of frequency bands.

In order to further prove the rationality of frequency band division in this study, we provide classification results of no band division, two bands ($F_{p,2}^{2,0}$ (0–32 Hz), $F_{p,2}^{2,1}$ (32–64 Hz)), four bands ($F_{p,3}^{3,0}$ (0–16 Hz), $F_{p,3}^{3,1}$ (16–32 Hz), $F_{p,3}^{3,2}$ (32–48 Hz), $F_{p,3}^{3,3}$ (48–64 Hz)) and eight bands. The relevant results are shown in Fig. 4. As can be seen, the average detection accuracy of multiple frequency bands exceeds 90%. Especially for eight frequency bands, the accuracy reaches 99.75%. It is worth mentioning that when no band division is performed, the classification accuracy is only 82.16%. This is because features in different frequency bands cannot be used specifically, resulting in information confusion. In summary, considering multiple frequency bands, the proposed method achieves excellent results in epilepsy detection and is expected to provide valuable reference for other EEG-based neurological disease detection.

## Methods

### Experiment validation

Taking the designed MMBN as input, the proposed AM-CNN model is trained and tested on the CHB-MIT dataset which was created and provided by Boston Children's Hospital (CHB) and the Massachusetts Institute of Technology (MIT) and included 24 subjects (5 males, 3–22 years old; 19 females, 1.5–19 years old).

All scalp electroencephalograms in the CHB-MIT dataset were collected using the international 10–20 system electrode placement method. The electrode positions used in the dataset were FP1, FP2, F7, F3, FZ, F4, F8, FT9, FT10, T7, C3, CZ, C4, T8, P7, P3, PZ, P4, P8, O1, O2, and the sampling frequency was 256 Hz. The dataset adopts a bipolar measurement method, where the collected EEG signals are recorded in the form of voltage differences between adjacent electrodes in a longitudinal direction. The number of electrode pairs (channels) contained in different subsets and different signal segments in the same subset varies from 18 to 23. In this paper, considering data integrity, we used 18 subjects, all of whom had 23 channels of EEG signals (involving frontal lobe: F3, F4, F7, F8, FZ; frontal lobe: FP1, FP2; temporal lobe: T7, T8; occipital lobe: O1, O2; parietal lobe: P3, P4, P7, P8; central lobe: C3, C4, CZ; ft9, ft10). Samples were segmented using a sliding window with a length of 1 s. The sliding step is 0.5 s. In this paper, we obtained 11,150 samples in total, including 5410 normal state samples and 5740 seizure state samples. Aim to avoid contingency, ten-fold cross validation is conducted. For one fold, 90% of the whole samples were selected as the training set and the remaining 10% as the test set. 10% of the training samples as the validation set. The model is trained via Adam optimizer with cross-entropy loss function. The best model is recorded when the loss on the validation set reaches a minimum value. The number of epochs is set as 200. Batch size is 64. Learning rate is set as 0.001. Three performance metrics, including accuracy, sensitivity, and specificity, are introduced to evaluate the model’s performance. All experiments are completed on workstation which is equipped with Intel CPU (i9-10920X, 3.5 GHz), NVIDIA GPU (RTX 3090), and 32 GB RAM.

### Multi-frequency multilayer brain network

We establish a multilayer brain network based on EEG signals to study epilepsy related brain states, where each layer corresponds to a specific frequency band. Taking *p*-channel EEG signals $\left\{ {x_{p,l} } \right\}_{{l{ = }1}}^{L} p = 1,2, \ldots ,N$, with a length of* L* as an example, MMBN is constructed as follows. Firstly, we perform 4-layer wavelet packet decomposition on the EEG signals of each channel to obtain sixteen frequency bands. The mathematical expression of wavelet packet decomposition is defined as:

8 $$\left\{ \begin{aligned} &F_{p,m}^{j + 1,2i} = \sum\limits_{l} {h\left( {m - 2n} \right)} F_{p,n}^{j,i} \begin{array}{*{20}l} {} &\,\,\,\,\,\,{\text{Approximate signal}} \\ \end{array} \hfill \\ &F_{p,m}^{j + 1,2i + 1} = \sum\limits_{l} {g\left( {m - 2n} \right)F_{p,n}^{j,i} } \begin{array}{*{20}l} {} & {\text{Detail signal}} \\ \end{array} \hfill \\ \end{aligned} \right.$$

where $F_{p,n}^{j,i}$ represents the sub-frequency band after *n*-layer wavelet packet decomposition of *p*-channel EEG signal. (*j*, *i*) is the node order of wavelet packet tree. $h\left( \cdot \right)$ is a low pass filter. $g\left( \cdot \right)$ is a high pass filter. *m* and *n* are the number of decomposition layers. The bandwidth of each frequency band is ${{\left( {{{f_{s} } \mathord{\left/ {\vphantom {{f_{s} } 2}} \right. \kern-0pt} 2}} \right)} \mathord{\left/ {\vphantom {{\left( {{{f_{s} } \mathord{\left/ {\vphantom {{f_{s} } 2}} \right. \kern-0pt} 2}} \right)} {2^{4} }}} \right. \kern-0pt} {2^{4} }} = 8\;{\text{Hz}}$, where $f_{s} = 256\;{\text{Hz}}$ means sampling frequency. Due to the fact that the frequency of EEG signals reflecting epileptic brain state is mainly distributed within 70Hz^42–44^, this study used eight frequency bands, including $F_{p,4}^{4,0}$ (0–8 Hz), $F_{p,4}^{4,1}$ (8–16 Hz), $F_{p,4}^{4,2}$ (16–24 Hz), $F_{p,4}^{4,3}$ (2432 Hz), $F_{p,4}^{4,4}$ (32–40 Hz), $F_{p,4}^{4,5}$ (40–48 Hz), $F_{p,4}^{4,6}$ (48–56 Hz), $F_{p,4}^{4,7}$ (56–64 Hz). The selected wavelet base is dbN, which has been proven by existing research to be able to decompose EEG signals and has fast computational speed^45, 46^.

Secondly, we use the function $wprcoef\left( \cdot \right)$ to reconstruct an approximation to raw EEG signals from selected nodes in the wavelet packet tree* T*. The signals for the reconstruction of sub-frequency band $F_{p,n}^{4,i}$ is

9 $$\left\{ {\overset{\lower0.5em\hbox{$\smash{\scriptscriptstyle\frown}$}}{x}_{p,l}^{4,i} } \right\}_{l = 1}^{L} = wprcoef\left( {T,F_{p,n}^{4,i} } \right)\begin{array}{*{20}c} {} & {i = 0,1, \ldots ,7} \\ \end{array}$$

Finally, in each frequency band, we define brain electrodes (or channels) as network nodes. The weight of edge between nodes $\kappa$ and $\nu$ is determined via the Spearman rank correlation coefficient. The mathematical expression is

10 $$w_{\kappa \nu }^{4,i} = 1 - \frac{{6\sum\limits_{l = 1}^{L} {\left[ {{\text{rg}}\left( {\left\{ {\overset{\lower0.5em\hbox{$\smash{\scriptscriptstyle\frown}$}}{x}_{\kappa ,l}^{4,i} } \right\}} \right) - {\text{rg}}\left( {\left\{ {\overset{\lower0.5em\hbox{$\smash{\scriptscriptstyle\frown}$}}{x}_{\nu ,l}^{4,i} } \right\}} \right)} \right]^{2} } }}{{L\left( {L^{2} - 1} \right)}}\begin{array}{*{20}c} {} & {\kappa ,\nu = 1,2, \ldots ,N} \\ \end{array}$$

where $${\text{rg}}\left( {\left\{ {\overset{\lower0.5em\hbox{$\smash{\scriptscriptstyle\frown}$}}{x}_{\kappa ,l}^{4,i} } \right\}} \right)$$ and $${\text{rg}}\left( {\left\{ {\overset{\lower0.5em\hbox{$\smash{\scriptscriptstyle\frown}$}}{x}_{\nu ,l}^{4,i} } \right\}} \right)$$ respectively represent the position of the $$l{\text{-th}}$$ element in the sequence $$\left\{ {\overset{\lower0.5em\hbox{$\smash{\scriptscriptstyle\frown}$}}{x}_{\kappa ,l}^{4,i} } \right\}$$ and $$\left\{ {\overset{\lower0.5em\hbox{$\smash{\scriptscriptstyle\frown}$}}{x}_{\nu ,l}^{4,i} } \right\}$$ after sorting.

Through determining the edge between each channel pair (or node pair) via the above method, the network under this frequency band can be obtained. In different frequency bands, the correlation characteristics between channels are significantly different, making it possible to obtain different frequency-dependent brain networks. By repeating the above process at eight frequency bands, a multilayer brain network can be constructed, which has eight layers with $N$ nodes per layer. For each layer, 30% of the edges with larger weights are reserved for subsequent analysis.

### Convolutional neural network model based on attention mechanism

**Figure 5:**
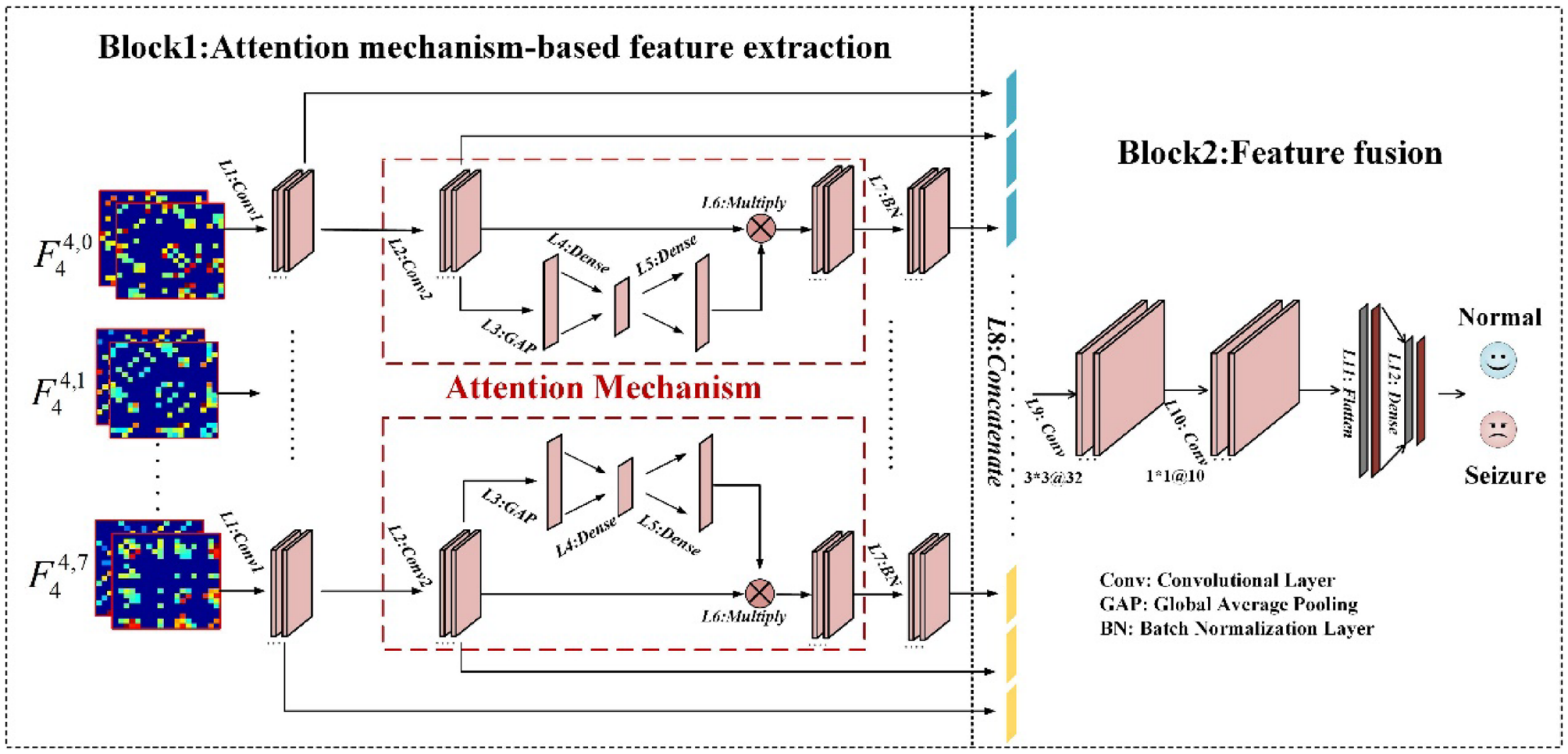
The architecture of the proposed AM-CNN model.

Here, take the obtained multilayer brain network as input, a convolutional neural network model based on attention mechanism (AM-CNN) is carefully designed for epilepsy detection. Figure 5 shows the detailed architecture. Table 4 lists the corresponding parameters.

**Table 4:** The parameters of the proposed AM-CNN model.

	Layers	Details	Output size
AM-FE	*L*1:Conv,	3 × 3@16, ReLU	23, 23, 16
	*L*2:Conv	3 × 3@16, ReLU	23, 23, 16
	*L*3:GAP		16
	*L*4:Dense	ReLU	8
	*L*5:Dense	Sigmoid	16
	*L*6:Multiply	*L*2 × *L*5, ReLU	23, 23, 16
	*L*7:BN		23, 23, 16
FF	*L*8:Concat		23, 23, 384
	*L*9:Conv	3 × 3@32, ReLU	23, 23, 32
	*L*10:Conv	1 × 1@10, ReLU	23, 23, 10
	*L*11:Flatten		5290
	*L*12:Dense	Softmax	2

Note:‘AM-FE’ means attention mechanism-based feature extraction module. ‘FF’ means feature fusion module. ‘Conv’, ‘GAP’, ‘BN’ and ‘Concat’ means convolutional layer, global average pooling layer, batch normalization layer and concatenate layer, respectively.

The AM-CNN model consists of two blocks. The function of the first block is a feature extraction based on the attention mechanism (AM-FE module), which is used to acquire multilayer network features from eight frequency bands. Note that, each layer of the multilayer brain network can be represented as an adjacency matrix, which is a grid like data. Elements at positions $\left( {\nu ,\kappa } \right)$ in the adjacency matrix represent weights $w_{\nu ,\kappa }$. The first module has eight branches, and it exactly matches the structure of the multilayer brain network. Each branch shares the same structure. Specifically, each branch is provided with two convolution layers (layer 1 and layer 2). Convolutional layer realizes feature extraction by designing a certain number of convolution kernels, which shows obvious advantages in processing grid-like data, such as the adjacency matrix here. In the following description, Layer 1 is simplified to *L*1, and others similarly. *L*1 and *L*2 can be described by the following formula:

11 $$\left\{ \begin{gathered} {{\varvec{\upsigma}}}_{k}^{1} = conv\left( {{\mathbf{w}}_{k}^{1} ,{\mathbf{A}}^{0} } \right) + b_{k}^{1} ,\,\,k = 1,2, \ldots K \hfill \\ {{\varvec{\upsigma}}}_{k}^{2} = conv\left( {{\mathbf{w}}_{k}^{2} ,{{\varvec{\upsigma}}}_{k}^{1} } \right) + b_{k}^{2} ,\,\,k = 1,2, \ldots K \hfill \\ \end{gathered} \right.$$

where ${\mathbf{A}}^{0}$ is the input data (i.e., adjacency matrix), ${{\varvec{\upsigma}}}_{k}^{1}$ and ${{\varvec{\upsigma}}}_{k}^{2}$ are $k{\text{-th}}$ output characteristic maps of *L*1 and *L*2, respectively. ${\mathbf{w}}_{k}$ and $b_{k}$ represent the weight matrix and deviation term of the $k{\text{-th}}$ convolution kernel, and $conv\left( \cdot \right)$ represents convolution operation. In each layer,$K = 16$ convolution kernels are designed, and the size is set to $3 \times 3$.

Due to the fact that convolution operations mainly handle local information of features. Directly processing the convolutional output features cannot effectively model the interrelationships between channels in the output features. To address this issue, we introduced channel-based attention mechanism after *L*2 to exploit the channel dependencies in outputs features ${{\varvec{\upsigma}}}^{2}$.

12 $${{\varvec{\upsigma}}}^{2} = \left[ {{{\varvec{\upsigma}}}_{1}^{2} ,{{\varvec{\upsigma}}}_{2}^{2} , \ldots ,{{\varvec{\upsigma}}}_{K}^{2} } \right]$$

where ${{\varvec{\upsigma}}}_{k}^{2} \in {\mathbf{\mathbb{R}}}^{H \times W}$ is the feature maps corresponding to the *k*-th convolutional kernel with the heights of *H* and widths of *W.* Specifically, in* L*3, channel-wise statistic ${{\varvec{\upsigma}}}^{3} = \left[ {\sigma_{1}^{3} ,\sigma_{2}^{3} , \ldots ,\sigma_{K}^{3} } \right]$ is generated by using global average pooling, and the *k*-th element $\sigma_{k}^{3}$ of ${{\varvec{\upsigma}}}^{3}$ is defined by

13 $$\sigma_{k}^{3} = \frac{1}{H \times W}\sum\limits_{\kappa = 1}^{H} {\sum\limits_{\nu = 1}^{W} {\sigma_{k}^{2} \left( {\kappa ,\nu } \right)} }$$

where $\sigma_{k}^{2} \left( {\kappa ,\nu } \right)$ is an element at the position $\left( {\nu ,\kappa } \right)$. In order to fully capture the dependencies between channels, we introduce two dense layers (*L*4 and *L*5) to form a bottleneck structure. The outputs of *L*5 is

14 $${{\varvec{\upsigma}}}^{5} = {\text{sigmoid}}\left( {{\mathbf{W}}_{2} \cdot {\text{ReLU}}\left( {{\mathbf{W}}_{1} {{\varvec{\upsigma}}}^{3} } \right)} \right) = \left[ {\sigma_{1}^{5} ,\sigma_{2}^{5} , \ldots ,\sigma_{K}^{5} } \right]$$

where ${\mathbf{W}}_{1} \in {\mathbf{\mathbb{R}}}^{{\frac{K}{r} \times K}}$ and ${\mathbf{W}}_{2} \in {\mathbf{\mathbb{R}}}^{{K \times \frac{K}{r}}}$ are the weight matrix of *L*4 and* L*5, respectively.*r* represents the reduction ratio, and the value of *r* is 2 in this study via trade-off between performance and computational cost. In *L*6, the output of *L*5 is used to weight each feature map of *L*2. The mathematical expression is

15 $${{\varvec{\upsigma}}}^{6} = \left[ {\sigma_{1}^{5} {{\varvec{\upsigma}}}_{1}^{2} ,\sigma_{2}^{5} {{\varvec{\upsigma}}}_{2}^{2} , \ldots ,\sigma_{K}^{5} {{\varvec{\upsigma}}}_{K}^{2} } \right]$$

In general, layers 3–6 constitute attention paths, which can enhance the effective features of* L*2.

*L*7 is a batch normalization (BN) layer that can mitigate overfitting and accelerate the training process. The implementation of BN is as follows:

16 $${{\varvec{\upsigma}}}_{k}^{7} = \theta {\hat{\mathbf{\sigma }}}_{k}^{6} + \pi$$

where $\theta$ and $\pi$ are learnable parameters. In order to ensure the distribution consistency of ${{\varvec{\upsigma}}}_{k}^{7}$ and ${{\varvec{\upsigma}}}_{k}^{6}$, ${\hat{\mathbf{\sigma }}}_{k}^{6}$ is the normalized input data having the following form:

17 $${\hat{\mathbf{\sigma }}}_{k}^{6} = \frac{{{{\varvec{\upsigma}}}_{k}^{6} - mean\left( {{{\varvec{\upsigma}}}_{k}^{6} } \right)}}{{std\left( {{{\varvec{\upsigma}}}_{k}^{6} } \right)}}$$

where $mean\left( \cdot \right)$ and $std\left( \cdot \right)$ represent the expected value and standard deviation of ${{\varvec{\upsigma}}}_{k}^{7}$.

The task of the second block is the feature fusion, which integrates the network features of multiple frequency bands, and then realizes the detection of epilepsy. Firstly, outputs ${{\varvec{\upsigma}}}_{k}^{1}$, ${{\varvec{\upsigma}}}_{k}^{2}$ and ${{\varvec{\upsigma}}}_{k}^{7}$ from eight frequency bands are concatenated together (*L*8). Then, we set a convolution layer (*L*9) with 32 kernels for feature fusion. The kernel size is $3 \times 3$. The 32 feature maps of *L*9 are learned through another convolution layer (*L*10), and the kernel size is $1 \times 1$. The outputs of *L*10 are flattened in *L*11. Finally, all learned features are input into a dense layer (*L*12) for epilepsy detection, using the softmax activation function.

## Data Availability

The data that support the findings of this study are openly available in CHB-MIT dataset at https://physionet.org/content/chbmit/1.0.0/.
